# Epidemiological and Evolutionary Outcomes in Gene-for-Gene and Matching Allele Models

**DOI:** 10.3389/fpls.2015.01084

**Published:** 2016-01-07

**Authors:** Peter H. Thrall, Luke G. Barrett, Peter N. Dodds, Jeremy J. Burdon

**Affiliations:** CSIRO Agriculture, Commonwealth Scientific and Industrial Research Organisation, CanberraACT, Australia

**Keywords:** metapopulation, disease resistance, pathogen infectivity, epidemiology, spatial structure, coevolution

## Abstract

Gene-for-gene (GFG) and matching-allele (MA) models are qualitatively different paradigms for describing the outcome of genetic interactions between hosts and pathogens. The GFG paradigm was largely built on the foundations of Flor’s early work on the flax–flax rust interaction and is based on the concept of genetic recognition leading to incompatible disease outcomes, typical of host immune recognition. In contrast, the MA model is based on the assumption that genetic recognition leads to compatible interactions, which can result when pathogens require specific host factors to cause infection. Results from classical MA and GFG models have led to important predictions regarding various coevolutionary phenomena, including the role of fitness costs associated with resistance and infectivity, the distribution of resistance genes in wild populations, patterns of local adaptation and the evolution and maintenance of sexual reproduction. Empirical evidence (which we review briefly here), particularly from recent molecular advances in understanding of the mechanisms that determine the outcome of host–pathogen encounters, suggests considerable variation in specific details of the functioning of interactions between hosts and pathogens, which may contain elements of both models. In this regard, GFG and MA scenarios likely represent endpoints of a continuum of potentially more complex interactions that occur in nature. Increasingly, this has been recognized in theoretical studies of coevolutionary processes in plant host–pathogen and animal host-parasite associations (e.g., departures from strict GFG/MA assumptions, diploid genetics, multi-step infection processes). However, few studies have explored how different genetic assumptions about host resistance and pathogen infectivity might impact on disease epidemiology or pathogen persistence within and among populations. Here, we use spatially explicit simulations of the basic MA and GFG scenarios to highlight qualitative differences between these scenarios with regard to patterns of disease and impacts on host demography. Given that such impacts drive evolutionary trajectories, future theoretical advances that aim to capture more complex genetic scenarios should explicitly address the interaction between epidemiology and different models of host–pathogen interaction genetics.

## Introduction

Host-parasite interactions are among the very best of biological systems in which to investigate the interplay of ecological and genetic factors leading to long-term coevolutionary associations (Ebert, 2008; Gomez and Buckling, 2011; Morran et al., 2012; Thrall et al., 2012). Recognition of the speed at which reciprocal responses may occur and the clear fitness consequences associated with such associations has stimulated considerable interest among evolutionary biologists in the long-term dynamics of plant and animal host-parasite interactions. This interest has come with an understanding that differences in the underlying genetic nature of antagonistic interactions can play a key role in generating divergent patterns of evolutionary change. However, most previous studies have focused on evolutionary change with relatively little investigation of how underlying genetic architecture may influence epidemiological dynamics and associated feedbacks. In this work we highlight how complexity in the genetic architecture of host-parasite interactions is critical for understanding coevolution and then use spatially explicit simulation modeling of MA and GFG scenarios to illustrate the importance of understanding how patterns of disease may relate to specific models of host–pathogen interaction genetics.

Theoretical and simulation modeling studies of evolutionary dynamics in host-parasite interactions have largely centered on two distinct [gene-for-gene (GFG) and matching allele (MA)] models to represent the basic genetic mechanisms underlying the interaction. As recently summarized by Dybdahl et al. (2014), a fundamental difference is that GFG interactions are based on the assumption that non-recognition (by the host) results in infection, while MA interactions assume that a match between pathogen and host genotypes is required for infection to be successful (Lively, 1999; Lambrechts et al., 2006). Analytical and simulation models based on these two scenarios or variants thereof, have investigated a range of questions regarding the evolutionary consequences of disease in natural systems (Frank, 1991a,b, 1992, 1993a; Damgaard, 1999; Fenton et al., 2009), recombination systems and the maintenance of sex (Agrawal and Lively, 2001; c.f. the Red Queen hypothesis; Hamilton et al., 1990; Parker, 1994), the importance of spatial scale in driving coevolution and patterns of local adaptation (e.g., Gandon et al., 1996; Thrall and Burdon, 1999, 2002; Nuismer, 2006; Tellier and Brown, 2011; Moreno-Gámez et al., 2013) and host shifts (Poullain and Nuismer, 2012). Some theoretical models indicate blurring of the conceptual boundaries in that shifts between MA/GFG evolutionary dynamics are relatively easy to achieve with only small changes in individual parameter values (Agrawal and Lively, 2002). As Dybdahl et al. (2008) stated, “there is little information available from coevolutionary theory to rule out alternative models,” which highlights the importance of using empirical data on the underlying mechanisms of host-parasite interactions to inform modeling choices.

Molecular studies are increasingly demonstrating a level of interaction complexity not envisaged in earlier studies of qualitative-based resistance systems (e.g., Dodds et al., 2006; Dybdahl et al., 2014). For example, in many systems there is evidence that infection processes involve multiple stages, with different stages varying with regard to the underpinning genetics involved. These may variously involve genetic interactions consistent with GFG, MA or quantitative models (Dybdahl et al., 2014). Some theoretical work has begun to explore the evolutionary consequences of such infection processes (e.g., Agrawal and Lively, 2003; Fenton et al., 2012). Overall though, as Lambrechts et al. (2006) note, much of the modeling work in this area has almost exclusively focused on issues to do with host–pathogen coevolution and the maintenance of genetic variation. As genetic characterisation of host–pathogen interactions becomes more sophisticated, it is of central importance that such knowledge be used to inform theoretical models to better predict patterns of disease prevalence and incidence in both natural and managed systems. Yet, despite the fact that the fitness consequences of different genetic assumptions is a major driver of coevolutionary trajectories (Salathé et al., 2008) and thus disease outcomes, to date, very little attention has been paid to the demographic and epidemiological consequences of different genetic models of infection.

Here we first briefly review the genetic basis of host resistance and pathogen infectivity in GFG and MA models. We focus on plant-fungal pathogen associations as it is these for which the most extensive empirical information is available. We then report on results from spatially explicit simulation models of MA and GFG interactions in which we track host demography, pathogen epidemiology and the evolutionary dynamics of host resistance and pathogen infectivity. Of particular interest was to examine how GFG or MA assumptions would alter pathogen persistence, and patterns of disease incidence (the percentage of local populations in which disease is present) and prevalence (the fraction of infected individuals within local populations where disease is present).

## The Genetic Basis of Disease Resistance in Plants

The genetic basis of disease resistance in plants has been the subject of a very large number of studies, particularly of cultivated species where breeding for disease resistance has been a key component of crop protection from disease. Resistance can broadly be categorized as either quantitative or qualitative with the genetic control of these two expressions of resistance being quite distinct.

### Quantitative Resistance

Quantitative resistance is controlled by the expression of many genes, each with small phenotypic effect. The relative resistance or susceptibility of different host lines typically remains relatively unchanged regardless of the aggressiveness of pathogen isolates. In a segregating cross between resistant and susceptible parents, quantitative resistance is manifest as a continuous range of phenotypes. This form of resistance is not the subject of either the GFG hypothesis or the matching allele model and will not be considered further here.

### Qualitative Resistance

Qualitative resistance on the other hand, is controlled by the expression of a limited number of genes with major phenotypic effects. Resistance is generally inherited in a Mendelian fashion with resistance usually being dominant to susceptibility. Typically such genes are effective against some pathogen isolates and not others thereby giving rise to variable patterns of host-specificity; furthermore, not all resistance alleles are fully expressed, resulting in some resistant reactions that still permit varying degrees of pathogen reproduction (e.g., partial resistance; Burdon, 1987; Burdon et al., 2014).

## The Genetic Interaction Models

### The Gene-for-Gene Hypothesis

Qualitative resistance lies at the heart of the GFG system elucidated by Flor in a series of elegant experiments involving the rust pathogen *Melampsora lini* and its host plant *Linum usitatissimum* (Flor, 1946, 1947, 1955). In essence, Flor (1951) found that “for each gene determining resistance in the host there is a corresponding gene in the parasite with which it specifically interacts.” In this scenario the occurrence of a resistance reaction is dependent on both the presence of genes for resistance in the host and the corresponding genes for avirulence (non-infectivity) in the pathogen. In a model single gene interaction, resistance is usually dominant [generating host phenotypes R_ (resistant) and rr (susceptible)] and infectivity is recessive [generating pathogen phenotypes V_ (non-infective) and vv (infective—referred to as “virulent” in the plant pathology literature)]. In multi-gene models the same basic principles apply. However, it is important to note that resistant reactions conditioned by a particular GFG combination are generally epistatic over susceptible reactions resulting from other combinations of host and pathogen genes. In other words, resistance at one locus will mask susceptibility at another.

Following Flor, there have been many studies of the genetic and molecular basis of qualitative resistance in plants and infectivity in associated pathogens, culminating in a detailed picture of plant immunity and the mechanisms pathogens use to evade such immunity (Jones and Dangl, 2006; Stergiopoulos and de Wit, 2009; Dodds and Rathjen, 2010; Barrett and Heil, 2012). In plant-pathogen associations the GFG model has been strongly supported by genetic data and most convincingly by the isolation and mechanistic understanding of genes governing plant immune responses to biotrophic and hemi-biotrophic pathogens (Dodds and Rathjen, 2010). All sequenced plant genomes contain a repertoire of several hundred genetically-encoded immune receptors whose role is to recognize specific components, usually effector proteins, of a number of different pathogen species and genotypes. In these systems plant immune receptors recognize specific pathogen components to trigger defense responses and this has become the most persuasive and widely used model for plant-pathogen coevolution (Brown and Tellier, 2011). The extensive variation that occurs in corresponding host and pathogen genes provides the basis for GFG type interactions between the host and its potentially numerous pathogens and parasites. These interactions have been molecularly defined in many different plant-pathogen interactions including bacterial, fungal, virus, oomycete, nematode pathogens and even insect pests, but particularly involve pathogens of a biotrophic or hemi-biotrophic nature that rely on living host cells for at least part of their infection cycle (e.g., rusts). Empirical support for GFG interactions has also been presented in some animal host-parasite systems (e.g., Wilfert and Jiggins, 2013).

Although it has been argued that GFG models have been largely based on agriculturally derived data that may be biased by breeding and other agronomic practices (Frank, 1996), extensive work in natural plant-pathogen systems makes this claim untenable. Indeed, the principal biological associations underpinning the molecular understanding of plant immunity involves natural bacterial and oomycete pathogens of the model (non-crop) plant *Arabidopsis thaliana* (e.g., Dangl and Jones, 2001; Chisholm et al., 2006; Van der Linden et al., 2013). Here, the molecular recognition events between numerous corresponding R and Avr proteins have been extensively defined (Mackey et al., 2002; Krasileva et al., 2010; Karasov et al., 2014; Le Roux et al., 2015; Sarris et al., 2015). Likewise, in the *Linum marginale—Melampsora lini* interaction, patterns of susceptibility and resistance to a wide range of pathogen isolates indicate the existence of multiple (>25) resistance genes or alleles, and pathogen isolates capable of attacking two or more resistance genes are commonly encountered (Lawrence and Burdon, 1989; Thrall et al., 2002; Barrett et al., 2008). In both *Senecio vulgaris* (groundsel) and *Lactuca serriola* (prickly lettuce), resistance to the pathogen *Golovinomyces fischeri* (* = Erysiphe fischeri)* is widespread and largely of a qualitative nature (Bevan et al., 1993a; Lebeda et al., 2013). In *S. vulgaris*, resistance is controlled by single or at the most two genes, and in a sample of 50 host lines a minimum of 14 resistance specificities were identified (Harry and Clarke, 1987; Clarke, 1997). In the pathogen population, isolates were identified that could overcome multiple resistance genes (Bevan et al., 1993b). Similarly, in the interaction between the pathogen *Phakopsora pachyrhizi* and *Glycine canescens*, patterns of resistance are consistent with GFG genetics (**Table 1A**; Burdon and Speer, 1984), and populations of *G. canescens* have been shown to contain 10 or more resistance genes with up to three present in individual host lines (Burdon, 1987). Evidence for GFG interactions has also been found in associations between multiple species of morning glory *(Ipomoea* spp.) and the rust pathogen, *Coleosporium ipomoeae* (Chappell and Rausher, 2011). In essence, in all these systems the challenge of multiple different host lines by multiple different pathogen systems typically results in a complex, highly asymmetric, two-dimensional matrix of resistant and susceptible infection types. Individual resistance genes may confer resistance to multiple different pathogen isolates carrying different combinations of avirulence alleles (for lists of examples see Thompson and Burdon, 1992; Parker, 1994).

**Table 1:** Patterns of host–pathogen responses in the three models. In all three cases “+” indicates the occurrence of a compatible reaction (susceptibility) while “-” indicates an incompatible reaction (resistance).

**(A)** Pattern associated with gene-for-gene interactions where R refers to host resistance loci and v is the recessive (infective) form of pathogen infectivity loci (i.e., V would be the non-infective form). Thus, v_1_ represents a pathotype able to infect completely susceptible hosts (---) or those with R_1_ only, but is ineffective against others assuming this pathotype also carries V_2_, V_3_, and V_4_.						

	**-**	**v**_**1**_	**v**_**2**_	**v**_**3**_	**v**_**1**_**v**_**2**_	**v**_**1**_**v**_**2**_ **v**_**3**_

---	+	+	+	+	+	+
R_1_	-	+	-	-	+	+
R_2_	-	-	+	-	+	+
R_3_	-	-	-	+	-	+
R_1_R_2_	-	-	-	-	+	+
R_4_	-	-	-	-	-	-
						

**(B)** Pattern associated with haploid matching allele interactions, assuming multiple alleles for hosts (H) and pathogens (P) at a single locus (see Agrawal and Lively, 2001 for tables showing diploid patterns).						

	**P**_**1**_	**P**_**2**_	**P**_**3**_	**P**_**4**_	**…P_n_**	

H_1_	+	-	-	-	-	
H_2_	-	+	-	-	-	
H_3_	-	-	+	-	-	
H_4_	-	-	-	+	-	
.						
.						
.						
H_n_	-	-	-	-	+	
							

**(C)** Pattern associated with the inverse matching allele model [row and column labels as for (b) above].							

	**P**_**1**_	**P**_**2**_	**P**_**3**_	**P**_**4**_	**…P_n_**	

H_1_	-	+	+	+	+	
H_2_	+	-	+	+	+	
H_3_	+	+	-	+	+	
H_4_	+	+	+	-	+	
.						
.						
.						
H_n_	+	+	+	+	-	


### The Matching-Allele Model

Although the theoretical basis of the MA model lies in conspecific recognition systems that distinguish self cells or tissues from non-self in animals (Agrawal and Lively, 2002), the concept of genetic recognition between host and parasite to allow infection is inherent in many disease systems. Many viruses for instance require recognition of specific host surface proteins to initiate infection (Lodish et al., 2000). As noted by Parker (1996), historically there has not been strong empirical support for the MA hypothesis, but a number of plant-pathogen associations have now been described where the underlying biological mechanisms conform to the MA assumptions (e.g., Oliver and Solomon, 2010). Thus, MA type interactions have been described in necrotrophic fungal pathogens of plants where toxin production in the pathogen and genes for sensitivity to those toxins in the host, result in interactions that essentially are mirror images of classic GFG interactions (Wolpert et al., 2002; Ciuffetti et al., 2010; Oliver and Solomon, 2010). Some plant viruses also show MA type interactions (Khalifa et al., 2012). Genetic evidence consistent with this model has also been found in some animal-pathogen interactions (Carius et al., 2001; Frank, 2002; Luijckx et al., 2013). For example, using detailed crossing studies, Luijckx et al. (2013) show that infection genetics in the *Daphnia-Pasteuria* system are consistent with MA assumptions.

Although the key conceptual feature that distinguishes MA models is that recognition leads to infection rather than resistance, there are also differences in the genetic architectures that have been implemented in the GFG and MA models. GFG models typically assume multiple genes with only two corresponding alleles in the host and pathogen, while MA models generally assume a single locus with multiple alleles in both partners. However this secondary difference has led to some confusion in the literature as there are two versions of the MA model that are essentially mirror images of each other, the matching and inverse matching allele models (Otto and Michalakis, 1998). The inverse MA model proceeds from essentially the same premise as GFG models, in that a genetic match (recognition) leads to incompatibility, but differs in assuming that an exact match between suites of host and parasite alleles is required for a successful immune response to occur (Nuismer, 2006). Both versions of the MA model have frequently been used in theoretical explorations of the potential for pathogens to favor selection for recombination in hosts (e.g., Jaenike, 1978; Hamilton, 1980; Bell, 1982; Otto and Michalakis, 1998; Agrawal and Lively, 2001) and in considerations of the evolutionary dynamics of plant-pathogen interactions (Frank, 1991a,b, 1993b, 1996; Gandon et al., 1996).

### Distinguishing GFG from MA Interactions

There has been a degree of confusion in the literature as to the identity of GFG, matching and inverse matching scenarios with models sometimes being inconsistently classified (as noted by Agrawal and Lively, 2001). Here we follow the notation used by Otto and Michalakis (1998) in a discussion of parasite mediated selection, and recognize the MA model as the one in which pathogen isolates with high fitness (inducing a susceptible response) on one host genotype (host and pathogen alleles are matching) are assumed to have low fitness (be non-infective) on all other host phenotypes (see also Dybdahl et al., 2014 for further discussion of the different interaction models). As a consequence, haploid or co-dominant diploid MA models are generally constrained such that pathogens carrying multiple infectivity genes cannot occur. For the haploid MA model, such a scenario results in the pattern of resistant and susceptible responses typified in **Table 1B**. More complex situations are possible in diploid situations if, for example, there is genetic dominance (Agrawal and Lively, 2001). In contrast, in the inverse matching allele model each pathogen isolate has high fitness on all but one host phenotype and no host carries multiple resistance genes. This results in the pattern of resistant and susceptible responses shown in **Table 1C**. More recently, Fenton et al. (2009) have introduced the inverse GFG concept which adds further possibilities, and may better reflect interactions in which parasites actively seek hosts.

In **Table 1**, patterns of resistance and susceptibility in the host–pathogen interaction matrix represent a key practical distinction between the GFG and both forms of the haploid MA model. Host–pathogen interaction matrices under the haploid matching or inverse matching allele scenarios always result in a symmetric matrix, while GFG matrices are highly asymmetric. However, because asymmetries can also occur in diploid versions of the MA model, the presence of asymmetry in infection matrices may not be a key distinguishing feature of the underlying genetics of a given interaction. The potential for universally infective pathogen isolates is a hallmark of GFG models, and sometimes lack of observation of such phenotypes is held as evidence against this model (e.g., Lambrechts, 2011; Evison et al., 2013). However, there is evidence of fitness costs associated with infectivity (Thrall and Burdon, 2003; Castagnone-Sereno et al., 2007; Montarry et al., 2010; Zhang et al., 2011), so this is not a strong inference. Moreover, spatial structure (e.g., metapopulations) can maintain variability in strict GFG situations, even without costs (Damgaard, 1999; Thrall and Burdon, 2002). When only a few host and pathogen genotypes are compared, chance selection of pathogen isolates and host lines may result in a pattern in which the models cannot be distinguished. However, as noted above, there is extensive empirical evidence for GFG interactions in plant-pathogen interactions, with the ability of particular pathogen isolates to overcome multiple resistance genes (singly or in combination) shown repeatedly in pathogen infectivity surveys and a range of plant breeding situations (e.g., Stakman et al., 1962; Luig, 1983; Crute, 1987; Thrall et al., 2001; Kolmer, 2005).

Nevertheless, real world scenarios are likely to be more complex than embodied in either the GFG or MA models and involve various combinations of the parameters assumed in each. For instance, the classic flax/flax rust system, which was the inspiration for GFG models, actually exhibits a combination of genetic parameters from both the GFG and inverse MA models (Dodds and Thrall, 2009). Multiple genes in the host and pathogen interact with recognition leading to incompatibility (as in GFG), but many of these loci are multi-allelic (as in inverse MA situations). Additional complexity comes from the fact that multiple Avr loci can interact with alleles of single resistance loci. In addition overlap in recognition specificities may occur such that some R gene alleles recognize more than one Avr gene allele, and vice versa. In some cases, GFG and MA interactions can involve the same genetic loci. For instance, *Xanthomonas* spp. produce a variety of transcription activator-like effectors (TALEs) that are delivered to host plant cells and activate transcription of certain genes that promote infection, such as sugar exporters (Boch and Bonas, 2010). Some host genotypes express resistance due to alterations in the TALE DNA binding sites that prevent induction of the target genes, giving rise to genetic interactions consistent with MA (or inverse GFG; Fenton et al., 2009) assumptions. However some plant genotypes also contain immune receptors that recognize these TALEs and induce resistance in a classical GFG manner. In other host genotypes, the TALE DNA binding site has been fused to a gene whose expression induces defense responses, again leading to GFG-type interactions although not based on immune receptor recognition in this case. Clearly, such complexity requires sophisticated models that can integrate different resistance mechanisms and genetic parameters, but particularly highlights the need to match evolutionary models to the observed interactions in particular host–pathogen systems.

## Patterns of Resistance and Infectivity in Natural Populations

In natural situations, host plant-pathogen associations typically occur as spatially structured assemblages of multiple individual demes each at least partially isolated from each other. Within these metapopulations, local host populations may vary from those that are highly susceptible to all pathogen isolates to those that are highly resistant (e.g., Thrall et al., 2001). Depending on the diversity of host resistance types present locally, associated pathogen populations often comprise a range of pathogen lines ranging in infectivity from very simple pathotypes capable of attacking only one host genotype to others with broad infectivity spectra that may overcome all, or virtually all, resistance present in the local host population (Burdon and Jarosz, 1992; Thrall and Burdon, 2003; Laine, 2007; Lebeda et al., 2008).

Careful genetic analysis of the basis of resistance in many of these interactions shows the qualitative nature of the resistance genes involved. Resistance is typically a dominant trait although, depending on the specific pathogen isolate—host line interaction, it may range in expression from a complete absence of pathogen colonization and growth to one in which some pathogen reproduction may occur (Burdon, 1994; Laine, 2007; Ericson and Burdon, 2009). Qualitative resistance genes frequently segregate independently and multiple segregating loci may occur within the same host line (e.g., up to three effective against *Phakopsora pachyrhizi* are present in individual lines of *Glycine canescens;* Burdon, 1987). This, coupled with the fact that single resistance genes can confer protection against multiple different pathogen isolates, results in an asymmetric relationship between host and pathogen lines, creating a complex matrix of resistant and susceptible responses. The often highly asymmetric nature of this matrix is well illustrated by the host–pathogen responses typically detected in any reasonable scale survey of such interactions (e.g., Lawrence and Burdon, 1989; Espiau et al., 1998; Niemi et al., 2006; Lebeda et al., 2008). These studies show that a broad range of responses are regularly encountered from host individuals with no detectable resistance, through individuals exhibiting various patterns of resistant and susceptible responses that depend on the specific identity of the interacting pathogen isolate, to hosts for which no corresponding infectivity genes have been detected. Equally importantly, viewed from the pathogen side of the equation, a broad selection of pathotypes exist ranging from those that can attack only single host lines through to those that carry multiple infectivity genes, and hence can attack many different resistant types. At the level of the individual population this may result in the presence of pathotypes capable of successfully attacking all host lines present locally (e.g., as seen in extensive surveys of wild *Melampsora lini* populations; Thrall et al., 2002).

## Why Does it Matter?

As Otto and Michalakis (1998) point out, “the results of any given host-parasite model will depend on the form of genetic interactions between hosts and parasites.’ From a theoretical perspective, a number of studies have shown that there can be significant qualitative differences in evolutionary outcomes of host–pathogen interactions driven by GFG versus MA genetics. For example, Nuismer (2006) showed that local adaption may be less likely to emerge in MA situations, particularly if diploid genetics are assumed as persistent cycles in genotype frequencies are much less likely to occur. Poullain and Nuismer (2012) also predicted that host shifts may be less likely under MA-type genetics than either IMA or GFG scenarios. On the other hand, Quigley et al. (2012) found that cooperation in bacteria-virus interactions is more likely to evolve under MA than GFG scenarios. In terms of coevolutionary dynamics, as discussed by Salathé et al. (2008), the potential for Red Queen dynamics is also quite sensitive to the underlying genetic assumptions.

A key point that has rarely been addressed is that the genetics of host resistance and pathogen infectivity are also likely to be of fundamental importance to pathogen persistence and patterns of disease prevalence and incidence. A broad understanding of the ecological and evolutionary dynamics of disease depends on both a clear picture of the molecular basis of host resistance and pathogen infectivity mechanisms and an integrated understanding of how ecological and genetic factors jointly generate and maintain these polymorphisms. From an applied perspective, such knowledge is likely to significantly advance our ability to slow or inhibit pathogen adaptation relative to hosts, and will contribute to the development of integrated approaches to disease management (Sapoukhina et al., 2009; Michelmore et al., 2013; Burdon et al., 2014; Zhan et al., 2014).

Given this, it is important to build models that provide an appropriate reflection of biological reality and therefore have potential to guide thinking about fundamental issues in pathology and host–pathogen coevolution as well as applied management of disease. Certainly the detailed genetic basis of resistance and infectivity is still poorly understood in animal host-parasite systems, and it is possible that in these situations the matching allele approach may provide a reasonable approximation to reality although evidence to support this contention is still relatively scarce (but see Luijckx et al., 2013). While in plant host–pathogen systems—the focus of this discussion—an overwhelming body of empirical studies, covering a wide range of host plants and pathogens with different life histories, broadly support the GFG model (as detailed above), it is also becoming increasingly clear that the genetics of real host–pathogen interactions are likely to be more complicated than encapsulated by the assumptions of either the GFG or MA models.

Nevertheless, comparison of the basic assumptions of the GFG and MA models highlights some differences that in turn have fundamental impacts on the predictions and outcomes of the models. Thus in single population (or non-spatial) GFG models where host and pathogen dispersal is essentially random, a dynamic polymorphism between resistant and susceptible hosts, and infective versus non-infective pathotypes, can only be achieved if there are fitness costs associated with infectivity and resistance (e.g., Leonard, 1969, 1977; Jayakar, 1970; Leonard and Czocher, 1980; Sasaki, 2000). Increasingly careful empirical assessments of potential fitness costs associated with the presence of either host resistance or pathogen infectivity have provided clear evidence for their existence in a range of plant and pathogen species (Brown, 2002; Thrall and Burdon, 2003; Tian et al., 2003; Korves and Bergelson, 2004; Barrett et al., 2011; Brown and Rant, 2013; Karasov et al., 2014). Such evidence implies that the presence of universally infective pathogen genotypes is not necessarily a critical test of the GFG model (Dybdahl and Storfer, 2003). In contrast, at least in haploid MA models, basic assumptions about the interaction between host and parasite automatically impose frequency-dependent selection (each host genotype can only be attacked by a single pathogen genotype, facilitating time-lagged changes in the relative abundance of different host and pathogen combinations in response to shifts in the selective advantage of currently common and rare genotypes) and provides an alternative explanation as to why “super races” might not dominate pathogen populations even in the absence of costs (but see Nuismer, 2006 for discussion of the diploid case where such cycles of frequency-dependent selection may be less likely to occur).

When GFG and MA models are extended to ecologically more realistic situations—spatially explicit metapopulations in which genetic drift, extinction, recolonization, localized gene flow and selection occurs—distinctly different predictions emerge concerning epidemiological and genetic outcomes. We demonstrate the extent of these different outcomes with a spatially-explicit two-dimensional simulation model in which a total of 5 resistance genes and 5 infectivity genes in all possible combinations were distributed among host populations occurring in a 100 × 100 grid array. The model includes both within and among population demographic processes (e.g., gene-flow and migration), as well as mutation and coevolutionary dynamics. Both host and pathogen dispersal distances can be varied to encompass the range of spatial structures seen in nature. In this interaction, the pathogen acts as a discrete lesion disease with no fitness costs being associated with either host resistance or pathogen infectivity, and host mortality being a function of disease severity (see Thrall and Burdon, 2002 for a full description of the model which assumes haploid genetics; we use this same model here but expand it to also include MA and IMA scenarios). By implementing different “rules” regarding the interaction of individual host and pathogen genotypes, we constructed three sub-models that simulated the consequences of (i) a classical GFG interaction; (ii) a matching allele interaction; and (iii) an inverse matching-allele interaction respectively. The demographic constraints imposed on host–pathogen dynamics by the inverse matching allele model (i.e., hosts are susceptible to the vast majority of the pathogens they encounter) were such that in a stochastic metapopulation setting pathogen persistence was very difficult to achieve. As a consequence this model is not considered further here.

**FIGURE 1:**
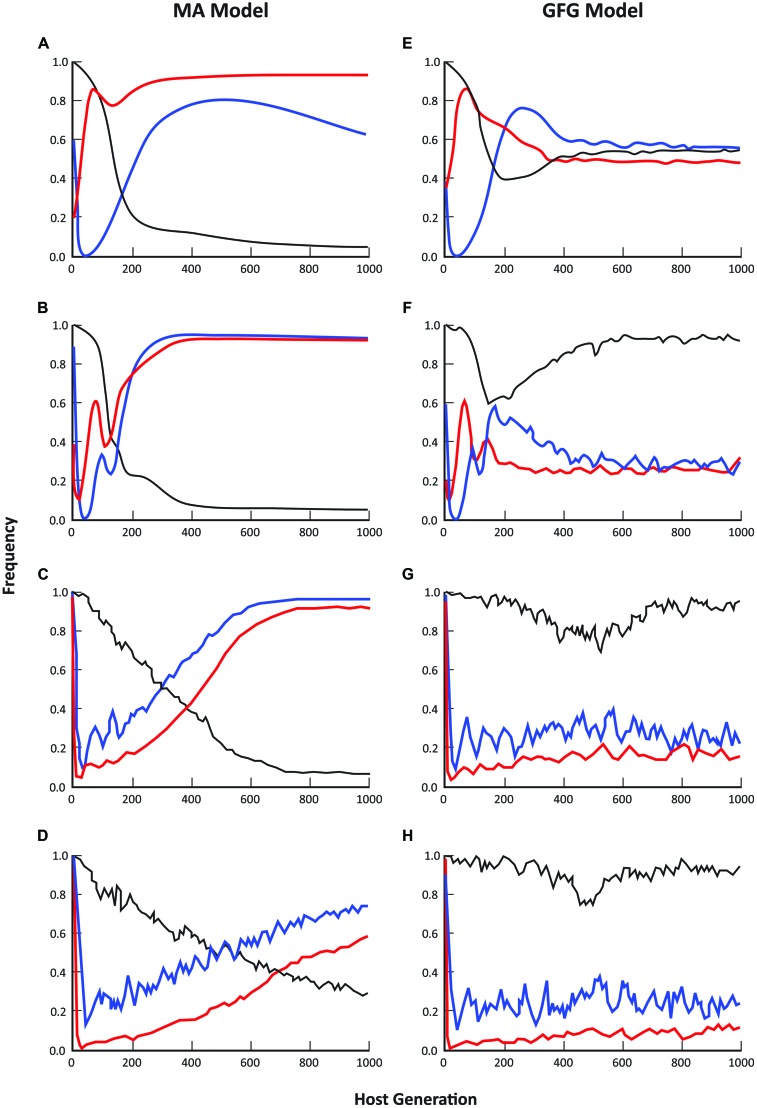
**Comparison of epidemiological patterns predicted by the matching allele and gene-for-gene models, where blue lines = disease incidence (% populations diseased), black lines = mean prevalence (% individuals infected in diseased populations), and red lines = % occupancy (fraction of sites with hosts present).** Each graph represents the mean of 20 simulation runs, where runs were randomly initiated with a subset of sites being occupied by hosts and pathogens. Moving from the top pair to the bottom pair of graphs, the maximum pathogen dispersal distance was set at 1 **(A, E)**, 2 **(B, F)**, 5 **(C, G)** and 10 **(D, H)** population units respectively. Host dispersal was fixed at 5 units. In all cases, simulations were initiated with a completely susceptible host and a non-infective (i.e., only infective on the susceptible host) pathogen and allowed to evolve over 1000 generations.

For both the GFG and MA models, dynamic patterns of average disease prevalence (within sites), the fraction of sites occupied by hosts and the fraction of those sites in which disease occurred, were strongly dependent on the spatial scale of pathogen dispersal (**Figure 1**). At pathogen dispersal distances of 2, 5, and 10 population units in the simulated metapopulation, the predicted equilibrium metapopulation structure for all these parameters showed considerable differences between the two models with disease incidence often approaching 100% in the MA model. Indeed, it was only at the smallest dispersal distance (*D* = 1) that the fraction of populations in which disease was present was similar under the two models (**Figures 1A,E**). Even then though, average disease prevalence and the number of sites occupied by hosts were very dissimilar with average prevalence across the metapopulation being considerably higher in the GFG model but with much lower levels of disease incidence than in the MA model (e.g., compare **Figures 1B,C**).

Empirical evidence from a wide range of natural host-fungal pathogen associations is in better accord with the epidemiological predictions of the GFG than the MA model. For example, in the *Filipendula—Triphragmium* (Burdon et al., 1995, unpublished) and *Valeriana—Uromyces* associations (Ericson et al., 1999), the number of populations infected with disease varies from year to year but is always substantially lower than the total number of host populations. Over an 11-year period, the proportion of populations of *Filipendula ulmaria* infected with the rust pathogen *Triphragmium ulmariae* ranged between 30 and 65% while the proportion of plants infected in those populations in which disease was present ranged from <1% to >90% (Smith et al., 2011; Ericson and Burdon, unpublished). In the *Valeriana—Uromyces* interaction the proportion of populations with disease present ranged between 40 and 78% over a 30 year period, while prevalence in infected populations ranged from 4 to 41% (Ericson et al., 1999; Ericson and Burdon, unpublished).

**FIGURE 2:**
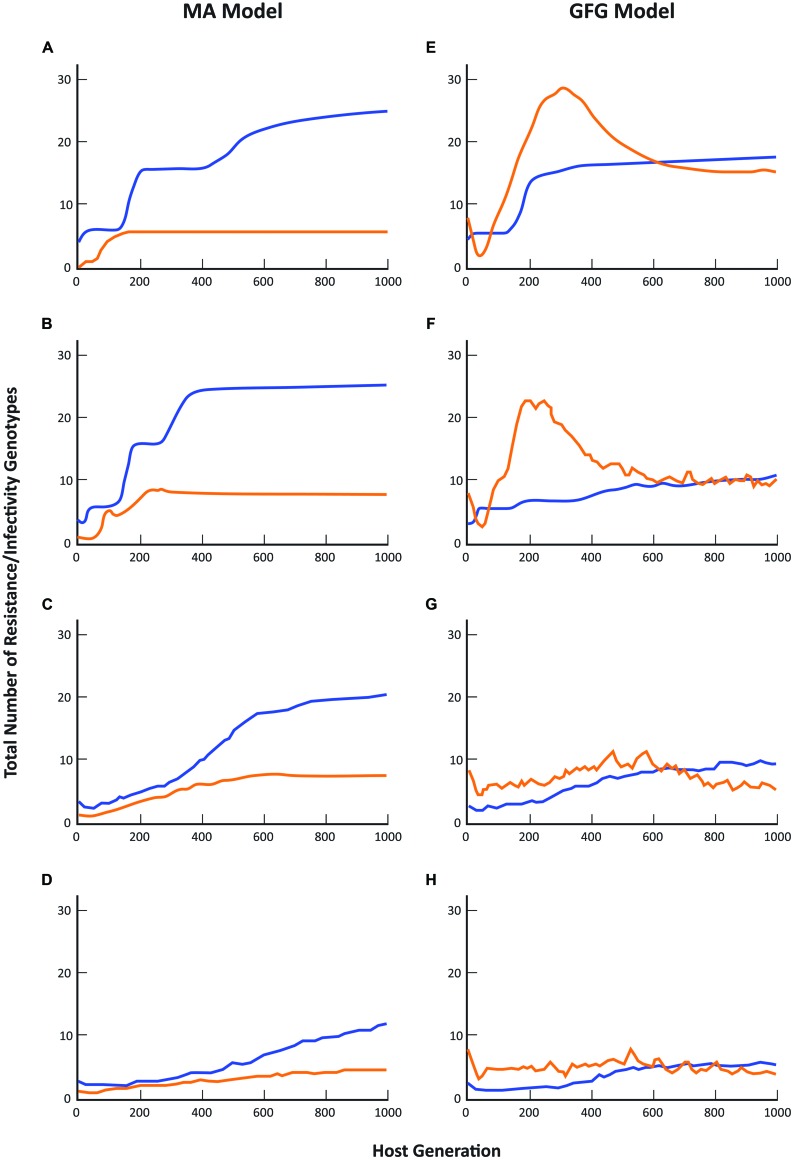
**Comparison of coevolutionary patterns predicted by the matching allele and gene-for-gene models.** Blue lines represent the total number of host resistance genotypes present across the metapopulation, while orange lines represent the total number of pathogen infectivity genotypes present. Simulation protocols are otherwise as described in **Figure 1** and in Thrall and Burdon (2002).

When genetic parameters are considered, the GFG and MA models again make substantially different predictions about the likely outcome of host-parasite co-evolutionary interactions although the effect of increasing spatial scale on overall diversity shows a generally similar trend toward lower numbers of both resistance and infectivity genotypes in the two models (see also Thrall and Burdon, 2002). Overall, the total number of resistance genotypes present under the MA scenario was always substantially greater than the total number of infectivity genotypes (**Figures 2A–D**). In contrast, in the GFG model, while the equilibrium structure indicated similar levels of diversity in both the total number of resistance and infectivity genotypes at all pathogen dispersal distances, dynamical patterns through time always showed periods when the numbers of infectivity genotypes exceeded resistance genotype numbers (**Figures 2E–H**).

These differences are perhaps not surprising given that under a GFG scenario new infectivity genes can evolve even when resistance is unchanged (e.g., many pathotypes can attack completely susceptible hosts, or indeed any particular resistance phenotype), while in the MA model, a pathogen cannot evolve until its host has first evolved (in other words until the host has gained resistance to that particular pathogen). As a consequence, it is never possible to get higher diversity in pathogens than in hosts. Note that the reverse situation is true for the inverse matching allele model where it is very difficult for host resistance to evolve as genetic changes can only have marginal effects on overall host susceptibility to the pathogen population. Detailed empirical evidence concerning spatial variation in the resistance and infectivity structures of co-occurring plant and pathogen populations is limited to just a few well-studied systems (Laine et al., 2011; Tack et al., 2012), but the evidence that does exist supports the general predictions of GFG models. Thus in the interaction between *Linum marginale* and *Melampsora lini*, pathogen diversity across an entire metapopulation is of the same order of magnitude as that of the co-occurring hosts (Thrall et al., 2001, 2002). In these instances we have been able to point to evidence that broadly supports the GFG model, at least for a range of natural plant-pathogen interactions that have been studied in some detail. Importantly, abundant genetic and molecular evidence of GFG-type interactions in a broad spectrum of plant-pathogen systems suggests that the GFG paradigm remains an important basis for theoretical and simulation models of plant-pathogen coevolution and disease dynamics. However, for many more systems, we lack detailed understanding of the genetics of resistance and infectivity. At the same time, for many questions regarding the epidemiology or evolutionary trajectories of host–pathogen associations in heterogeneous environments, the consequences of different biological assumptions regarding the genetics of host–pathogen interactions have not been well characterized (Kwiatkowski et al., 2012). This is crucial, given that even for simple parameters, qualitatively different predictions emerge from different models.

## Conclusion

From a theoretical perspective, the genetics of host–pathogen interactions as represented by GFG and MA formulations can be viewed as endpoints of a continuum (e.g., as suggested by some models; Agrawal and Lively, 2002). However, biologically, while combinations of the two are clearly possible, recognition must either lead to compatibility or incompatibility. Empirical studies of real world systems increasingly highlight the complexities of interactions at the molecular level (multi-stage infection which can mean GFG-like processes operating at one stage and MA-like at another; e.g., Fenton et al., 2012). A central point of the brief modeling exercise we have presented here is that characterizing the nature of genetic interactions is important—there are distinct epidemiological as well as evolutionary consequences that follow from different genetic assumptions (Dybdahl et al., 2014).

Not only do host–pathogen interactions in the real world vary along the MA-GFG continuum, but the importance of infectivity and resistance costs and the role of spatial structure in driving these interactions will also vary in relation to host and pathogen life history. Moreover, recent work suggests that multi-step infection processes are likely to be an important consideration in many systems—both the nature of the genetic interactions and the potential for coevolutionary dynamics may vary at different stages. It is currently unclear whether phenotypic patterns of infection and resistance (e.g., the degree of asymmetry, the presence of “super-infective” pathotypes) are likely to be good indicators of interactions at a genetic level. Rather than debating whether MA or GFG formulations are more realistic (there is evidence for both), we need to focus on the epidemiological and evolutionary consequences of different assumptions. At the same time, there is enormous intellectual value in such discussions (analogous to productive debates in past decades on density vs. frequency-dependent disease transmission or the ecological importance of density-dependent vs. density-independent processes).

One goal of future research should be to better predict the consequences of different genetic structures for disease spread and persistence in real-world systems and the follow-on implications for management. This obviously presents a challenge in systems where family level genetic data is difficult or impossible to obtain. Network-based topological approaches (see Barrett et al., 2015 and references therein for examples) and model-based statistical inference (Heath and Nuismer, 2014) offer potential means for inferring the underlying genetics from population-level information about host–pathogen interactions or patterns of disease. Currently, we lack a comprehensive understanding of how phenotypic and population level predictions of disease may vary in relation to different underpinning genetic models of infection. This is increasingly recognized as being of particular importance with respect to managing the epidemiology and evolutionary potential of pathogens in agro-ecological systems (Burdon et al., 2014; Mundt, 2014; Zhan et al., 2014; Papaix et al., 2015). Finally, the complexity of host–pathogen interactions in the real world is far greater than is typically modeled (Engelstädter, 2015). It would be useful to determine to what extent, if any, different kinds of interactions can be categorized according to an underlying genetic model.

## Author Contributions

PT, JB conceived the ideas for the study and the model framework. PT developed and ran the simulation model. PT, JB analyzed and interpreted the simulation data. All authors contributed substantially to writing and reviewing the paper.

## Conflict of Interest Statement

The authors declare that the research was conducted in the absence of any commercial or financial relationships that could be construed as a potential conflict of interest.
